# Effectiveness of Epley–Canalith Repositioning Procedure versus Vestibular Rehabilitation Therapy in Diabetic Patients with Posterior Benign Paroxysmal Positional Vertigo: A Randomized Trial

**DOI:** 10.3390/life13051169

**Published:** 2023-05-12

**Authors:** Mohammad Abu Shaphe, Mohammed M. Alshehri, Ramzi Abdu Alajam, Rashid Ali Beg, Najat Ibrahim A. Hamdi, Saravanakumar Nanjan, Vandana Esht, Mohammed A. Aljahni, Hashim Ahmed, Ausaf Ahmad, Aafreen, Ashfaque Khan, Abdur Raheem Khan

**Affiliations:** 1 Department of Physical Therapy, College of Applied Medical Sciences, Jazan University, Jazan 45142, Saudi Arabia; 2 Physical Education Department, Jazan University, Jazan 45142, Saudi Arabia; 3 Department of Medical Rehabilitation Science, College of Applied Medical Sciences, Najran University, Najran 66252, Saudi Arabia; 4 Department of Community Medicine, IIMS&R, Integral University, Lucknow 226026, India; 5 Department of Physiotherapy, Integral University, Lucknow 226026, India

**Keywords:** balance, benign paroxysmal positional vertigo, diabetes mellitus, rehabilitation

## Abstract

Benign paroxysmal positional vertigo (BPPV) is a common inner ear disorder, characterized by brief episodes of vertigo caused by changes in head position. The condition can cause significant functional impairment and reduced quality of life. BPPV is especially common among diabetic patients. The Epley–canalith repositioning procedure (CRP) and vestibular rehabilitation therapy (VRT) are two commonly used interventions for the treatment of BPPV. The objective of this study is to compare the effectiveness of Epley–canalith repositioning procedure (ECRP) and Vestibular Rehabilitation (VR) therapy in the management of vertigo among Type 2 Diabetes Mellitus patients. A total of 30 subjects with Type 2 diabetes mellitus, aged between 40 and 65 years, were randomly allocated to either the ECRP or VR therapy groups using a lottery method, and then underwent Epley–canalith repositioning procedure or vestibular rehabilitation therapy, respectively. The outcomes measured by the study were Vertigo Symptom Scale–Short Form (VSS–sf) score and Berg Balance Scale (BBS) score, assessed pre-treatment (pre) and 4 weeks post-treatment (post). The results demonstrated that both ECRP and VR therapy led to improvements in VSS–sf and BBS scores. However, VR therapy was found to be more effective, resulting in a 13.6% higher improvement in VSS–sf scores (*p* = 0.03) and a 5.1% higher improvement in BBS scores (*p* = 0.51) compared to ECRP. Both Epley–canalith repositioning procedure and vestibular rehabilitation therapy are effective in managing BPPV in diabetic patients. Although the differences in BBS scores are not statistically significant, VRT demonstrated a trend towards greater improvement. Vestibular rehabilitation therapy can be used by clinicians as another rehabilitation technique for improving vertigo, postural stability, and activity of daily living in diabetic patients with BPPV.

## 1. Introduction

Benign paroxysmal positional vertigo (BPPV) is a common vestibular disorder characterized by intermittent, characterized by repeated bouts of vertigo that are triggered by changes in head position. BPPV can result from the dislodgement of otoconia, calcium carbonate crystals in the inner ear, which interfere with the normal sensing of head position and movement [1]. It is estimated that BPPV affects up to 2.4% of the general population, with increasing prevalence among elderly individuals [2]. Furthermore, diabetic patients have been reported to be at a greater risk of developing BPPV than non-diabetic individuals [3].

Diabetes mellitus type 2 (DMT2) is anticipated to increase in prevalence in the adult population worldwide from 463 million (9.3%) in 2019 to 700 million (10.9%) in 2045 [4]. Uncontrolled glycemia can lead to micro- and macro-vascular alterations, as well as sensory and motor system dysfunctions, in the long run. This can result in a loss of balance and a higher likelihood of falling. In order to maintain balance and correct posture, the human body needs to integrate inputs from the visual, somatosensory, and vestibular systems. During the execution of activities, these mechanisms modify destabilizing forces and preserve vertical posture of the body. DMT2 can impact negatively any of these three sensory systems.

The process of proprioceptive sensory information for optimal balance regulation may be compromised when vision and somatosensory function are disturbed. The vestibular system is crucial to this control, and its failure has been recognised as a separate fall risk factor. It has been discovered that patients with diabetes are 70% more likely to suffer inner ear impairment than those who do not. BPPV (benign paroxysmal positional vertigo) is more common in diabetic individuals (46%) than in people without diabetes (37%) [5]. Furthermore, people with diabetes and vestibular symptoms have a 12-fold higher risk of falling than people without symptoms.

Treatment for BPPV typically involves physical maneuvers, such as the Epley–canalith repositioning procedure (ECRP) and vestibular rehabilitation therapy (VRT), which aim to resolve the underlying cause of the vertigo [6]. The ECRP involves a series of head movements designed to guide the displaced otoconia out of the affected posterior semi-circular canal, whereas VRT is a customized exercise program that aims to improve the vestibular compensation process [7].

Diabetes can impair the vestibular system through microvascular changes, neuropathy, and oxidative stress, which might result in a higher persistence of BPPV symptoms and a reduced response to treatment [8,9]. Therefore, it is crucial to understand the effectiveness of ECRP and VRT in diabetic patients with BPPV.

To our knowledge, there is no prior study that explicitly compares the effectiveness of ECRP and VRT in diabetic patients with BPPV. The findings of this study could be quite insightful in terms of optimal management of BPPV in this population.

The study aims to determine which treatment is more effective in reducing the severity and frequency of vertigo symptoms, improving balance, and reducing the risk of falls. Understanding which treatment is more effective can help clinicians make more informed decisions about which treatment to recommend to their patients. The findings of this study will provide crucial details as to the most effective method for treating BPPV in diabetic individuals. This can improve their quality of life and lower the cost of managing the disease.

## 2. Materials and Methods

### 2.1. Sample Size Calculation

The study was conducted after receiving approval from the Institutional Ethical Committee of Jazan University, Jazan, Saudi Arabia. The trial is registered under Clinical Trials.gov with registration number NCT05828433. This study used a convenient sample of the population that was selected using purposive sampling. The G. Power 3.15 software (Franz F, Universität Kiel, Kiel, Germany) was used to calculate the number of subjects based on net improvement. Expecting at least 2% improvement (i.e., change from pre to post) between the two groups (Epley–canalith repositioning procedure and vestibular rehabilitation therapy) with regard to outcome measurements (VSS–sf score and BBS score) and considering 5.0% margin of error (Type I error) and 80.0% power (Type II error), a minimum of 30 patients/subjects were needed in the sample (*n*).

### 2.2. Subject

Thirty subjects were recruited on the basis of inclusion and exclusion criteria. Patients who were diagnosed with posterior canal BPPV by an ENT physician and diabetes mellitus (Type 2) by a physician through laboratory investigations, both male and female, aged between 20 and 70 years, possessing a positive Dix–Hallpike test, with nystagmus lasting less than 60 s, who were willing to participate in the study, and with a minimum score of 25/56 in the Berg Balance Scale were included. Conversely, patients taking antivertigo drugs, who had been treated for a similar vertigo experience, who had a disease of different origin that may have caused vertigo-like migraines (e.g., multiple sclerosis, stroke, traumatic brain injury), who had undergone CRP before, who had other causes of peripheral vertigo such as Meniere’s disease, vestibular neuritis, labyrinthitis or perilymphatic fistula, or who had pathologies contraindicated for Dix–Hallpike manoeuvre (e.g., prolapsed intervertebral disk, cervical spine instability, cervical myelopathy, previous cervical spine surgery) were excluded. All subjects provided written informed consent, and study procedures were carried out in conformity with the 1964 Helsinki Declaration.

### 2.3. Procedure

The participants were randomly divided into Group A for Epley–canalith repositioning procedure and Group B for vestibular rehabilitation therapy using a lottery system. A box contained 40 folded sheets of the same size and form, that were labelled either Group A (20) or Group B (20). The paper drawn by the patients allocated their mode of treatment. The group allocation was concealed, and the assessor was blinded. After group allocations, respective subjects were treated either with Epley–canalith repositioning procedure or vestibular rehabilitation therapy. Both treatments were given as individual treatment by the same physiotherapist for 4 weeks, and reassessment was carried out after 4 weeks. Before randomization, the baseline characteristics of the two treatment groups, including age, weight, height, BMI (body mass index), and Dix–Hallpike test, were evaluated. In a similar manner, outcome variables, including the Vertigo Symptom Scale–sf and Berg Balance Scale, were also evaluated by the same tester and physiotherapist supervising the test procedure at the baseline (day 0), as well as at the end of the treatment (after 4 weeks).

Test and re-test of the two groups were conducted in the same place with the same environment. Prior to the experiment, all subjects received thorough instruction on the measurement variables and their results. The subjects were also informed about the experimental risks, if any. All subjects were permitted to take treatment for their diabetes under the supervision of their consultant physician. No other treatment for vertigo symptoms was allowed during the study period, other than the Epley–canalith repositioning or vestibular rehabilitation therapy.

### 2.4. Interventions

#### 2.4.1. Epley–Canalith Repositioning Procedure (Group A)

In Group A, the affected posterior canal (the posterior semi-circular canal of the under-most ear when the nystagmus is provoked) was predetermined by Dix–Hallpike test. The time of latency and duration of induced nystagmus were recorded by an assistant using a stopwatch. This provided an estimation of the time it would take for the canalith bolus to complete a 90-degree rotation. Patients were informed about the procedure before the intervention. To monitor the nystagmus, the patients were instructed to keep their eyes open. In order for the patient’s head to extend beyond the edge of the examination table when in the Hallpike posture, the patient was seated lengthwise on the examination table. An assistant was standing by the side of the affected canal. The following steps were performed [9,10]:

- Step 1: The patient was brought down with the head tilted 45 degrees towards the affected canal, as in Hallpike test. The neck was extended;
- Step 2: The head was rotated 90 degrees towards the unaffected side. The neck was extended;
- Step 3: The head and body were rotated further by 90 degrees from the previous position (now facedown). The neck was in a neutral position;
- Step 4: The patient was brought into a sitting position while having their head turned constantly in the direction of the unaffected side’
- Step 5: The head was turned forward and the chin was kept down by 20° for a minute.

Since all the patients had nystagmus lasting for less than 60 s, each position was maintained for 60 s. The process was continued until there was no longer any nystagmus during the previous cycle or until no improvement was shown between the previous two cycles. The intervention was given for approximately 15 min, for 2 times per week, for 4 weeks.

Before going home, all the patients were given the following instructions: (1) The patients were asked to wait for 10 min after the manoeuvre was performed before going home; (2) Patients were instructed not to lie supine and to keep their head at 45-degree reclining positions while sleeping for 2 days; (3) All patients were asked to avoid provoking head positions such as bending over or looking up or down for 7 days following the procedure.

#### 2.4.2. Vestibular Rehabilitation Therapy (Group B)

Group B received vestibular rehabilitation therapy, which consisted of habituation exercises, gaze stability, and balance training.

Habituation exercises: The patient was asked to position themselves on the side of the examination table. Then, he/she was warned that the exercises could worsen the frequency and intensity of vertigo in the beginning, but that they should not alarmed because the symptoms would subside with the continuation of the intervention. Each manoeuvre in this experiment was carried out passively. Vertigo elicitation was reported for each measurement (M+ or M−). Intensity and duration of vertigo was recorded. Nystagmus occurrence was determined, and either its presence or absence was recorded (Ny+ or Ny−). In the case of exacerbation of symptoms, the exercises were modified by decreasing the repetition or stopping the exercises until the symptoms disappeared. The frequency and duration of the exercises were customized according to the patient’s response to the exercises. In this study, the patients were asked to repeat the exercises 5–10 times for 5 to 10 min, for 2 days per week, for 4 weeks. The exercise programs were graded so that the patient progressed from easily tolerated movements to difficult ones. The protocol for habituation exercises is shown in Table 1.

Gaze Stability Exercises and Balance Training [13]: The exercises progressed from sitting to standing, and support surface conditions were systematically varied progressing, from firm surface to compliant and regular surfaces. The exercises were performed in 3 sets or 5 repetitions, for 2 days per week, for 4 weeks. The full list is shown in Table 2.

### 2.5. Outcome Measurements

#### 2.5.1. Vertigo Symptom Scale–Short Form

VSS, consisting of 36 items, assesses frequency and severity of dizziness symptoms within the last 12 months. Both a long (VSS–lf) and a short form (VSS–sf) are available. There are 15 items in the VSS–sf. Each response is graded on a 5-point scale (0–4): 0 represents never, 1 represents a few, 2 represents several, 3 represents rather frequently, and 4 represents very frequently. The item scores are added up to determine the severity of the symptoms. The entire scale score is between 0 and 60. A higher score denotes a more serious issue. The scale consists of two subscales: 7 items measuring autonomic anxiety symptoms (VSS-A, score ranging from 0–28), and 8 items measuring vertigo–balance (VSS-V, score ranging from 0–32). The VSS–sf has proven to be a reliable and valid tool for evaluating vertigo patients. The VSS–sf exhibits high internal consistency (alpha = 0.9), and r = 0.52 is the construct validity. It has high test–retest reliability, with ICCs of VSS–sf = 0.88, VSS-V = 0.90, and VSS-A = 0.90 [14].

#### 2.5.2. Berg Balance Scale

BBS is used to test if a patient can securely balance themself while doing a series of predetermined tasks. It consists of 14 items, each of which has a 5-point ordinal scale, ranging from 0 to 4, with 0 denoting the lowest degree of function and 4 denoting the highest level. It takes about 15 to 20 min to complete. The maximum Berg Balance Scale score is 56, and it is divided into four ranges: 45–56 is for those who are more independent in their daily living and carrying the lowest risk of fall, 41–44 means patients are independent in their movement but carry some significant risk of fall, 21–40 means patients may ask for assistance while performing some balance tasks and, in general, there is 100% risk of fall, and 0–20 means that the patient requires a wheelchair and has a 100% of risk of fall. Berg Balance Scale is a reliable and valid assessment tool. The relative intra-rater reliability of the BBS with a pooled estimate of 0.98 has 95% CI 0.97 to 0.99, and the relative inter-rater reliability of the BBS with a pooled estimate of 0.97 has 95% CI 0.96 to 0.98 [15].

## 3. Results

**Figure 1:**
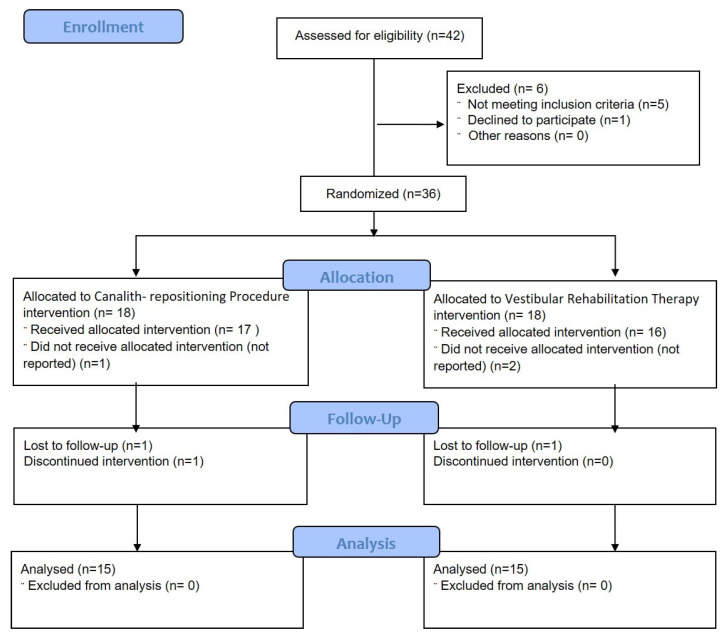
Flow diagram depicting the number of patients in each treatment group.

A total of 36 patients were assigned, out of 42 patients enrolled, from Jazan University Hospital, January 2022 to January 2023. Six patients dropped out for various reasons (see Figure 1). Figure 1 shows that, of the 30 patients who successfully finished the study, 15 underwent Epley–canalith repositioning procedure (Group A) and 15 underwent vestibular rehabilitation therapy (Group B). These 30 patients had a mean age of 39.40 ± 3.29 years.

### 3.1. Statistical Analysis

Data were summarized as Mean ± SE (standard error of the mean). In order to assess the normality of the distribution of our data, we performed the Kolmogorov–Smirnov test. This non-parametric test allowed us to determine if our data conformed to a normal distribution. A paired *t* test was used to compare the pre and post groups. The independent Student’s *t* test was used to compare the pre to post change (pre–post or post–pre) in the outcome measurements of the two independent groups. Discrete (categorical) data were summarized in number (*n*) and percentage (%), and the chi-square (χ^2^) test was used to compare them. Statistical significance was determined by a two-tailed (α = 2) test, *p* < 0.05. SPSS programme was used for analysis (Windows version 17.0).

### 3.2. Demographic Characteristics

Table 3 summarises the demographic characteristics of the two groups at the baseline level. The baseline demographic features of the two groups were similar when compared (*p* > 0.05) (Table 3), indicating that the groups were demographically similar, and that these parameters would not have an impact on the results.

### 3.3. Outcome Measurements

The pre and post outcome measurements of the two groups are summarised in Table 4. The VSS–sf score and BBS score improved significantly (*p* < 0.001) when the outcome measurements of each group were compared over the time periods.

### 3.4. Net Improvement

The pre-to-post changes in outcome measurements were carried out and compared using an independent Student’s *t* test, summarised in Table 5, to determine the efficacy of one group compared to the other. After 4 weeks, the results showed significant improvement in VSS–sf score (*p* < 0.05) of Group B as compared to Group A. However, BBS score did not (*p* > 0.05) show significant improvement of one group over the other group i.e., they were found to be statistically the same.

## 4. Discussion

Benign paroxysmal positional vertigo (BPPV) is a common vestibular disorder that affects individuals of all ages, including those with diabetes. The Epley–canalith repositioning procedure (ECRP) and vestibular rehabilitation therapy (VRT) are two commonly used treatments for BPPV. A randomized trial was conducted to compare the effectiveness of these two treatments in diabetic patients with BPPV. This study found that both Epley–CRP and VRT were effective in improving the balance and vertigo of diabetic patients with BPPV. However, VRT was found to be more effective in improving Vertigo Symptom Scale–short form (VSS–sf) scores of patients. This indicates that VRT could be a more appropriate treatment option for diabetic patients with BPPV.

Previous studies have shown that VRT is effective in enhancing balance and quality of life in BPPV patients. A meta-analysis of 20 randomized controlled trials found that VRT was beneficial in enhancing balance and minimizing dizziness in patients with BPPV [16]. Another study discovered that VRT effectively enhanced the balance and quality of life of elderly patients with BPPV [17].

The effectiveness of VRT in diabetic patients with BPPV may be due to several factors. Diabetes is known to cause peripheral neuropathy and impaired proprioception, which can affect balance and contribute to falls [18]. VRT targets these underlying balance deficits by using exercises that improve proprioception and strengthen the muscles involved in balance control. Additionally, VRT can be tailored to individual patients, which may improve treatment outcomes [19]. In contrast, Epley–CRP may not be as effective in diabetic patients with BPPV because it targets only the mechanical problem of displaced otoconia in the inner ear. It does not address the underlying balance deficits that may be present in diabetic patients. Additionally, Epley–CRP may be contraindicated in some patients with diabetes due to comorbidities such as cervical spine problems and obesity [20].

The improvement in Group A is supported by a study performed by Bahadir et al., in which 77% of the participants showed complete healing after one or two sessions of ECRP. Their immediate cure rate was 30.7% after the first manoeuvre [21]_._ In addition, Cohen-Shwart et al., showed that BPPV patients have impaired postural control relative to individuals with healthy lives, where vestibular system function was the primary determinant of balance. In those individuals with a successful ECRP, the balance score observed at baseline improved after the intervention and those scores were similar to those of the healthy participants [22].

In this study, CRP was proven to be effective, as it successfully moved the displaced canalith from the semi-circular canal to the vestibule from which they were absorbed, to stop the false signals and the debilitating symptoms of BPPV. A study also demonstrated that CRP is an effective, safe, quick, inexpensive procedure for patients with posterior canal BPPV. It is immediately effective and can be practiced without the need for sophisticated gadgets. It also stated that change of position during this procedure should be done gradually, as it is better tolerated by the patients [10].

Moreover, the improvement in the scores of VSS–sf and BBS, which suggest that vestibular rehabilitation therapy is effective in treating diabetic patients with PC BPPV, is supported by the study conducted by Deshpande et al., which concluded that VRT is a well-established and accepted intervention for persons with balance and vestibular disorders. The authors also described VRT as effective in decreasing dizziness and improving functional independence [23,24].

Several studies have demonstrated that persons with vestibular disorder may suffer fear of movement and psychologic dysfunction the longer they experience vestibular symptoms [25]. Early treatment is advised, as VRT has been shown to improve vestibular symptoms faster than no treatment. Bressi et al., also demonstrated that VRT is a simple, effective, low-cost treatment method for promoting visual stabilization, expanding static and dynamic postural stability, and enhancing the ability to maintain balance, quality of life, and vestibular–visual interaction during motion of the head in patients with BPPV [26].

Even though Epley–canalith repositioning was proven to be effective in this study from analysis within the group, this procedure was proven to be less effective when compared to vestibular rehabilitation therapy. Indeed, the residual symptoms may remain even after the disappearance of typical vertigo and nystagmus following successful Epley–canalith repositioning [27]_._ However, it is generally known that BPPV has a high recurrence rate, and few long-term follow-up studies have been conducted following particle repositioning manoeuvres, possibly as a result of the great short-term outcomes. While CRP is not taught to patients in the same way as VRT, it is possible to propose that free-floating canaliths should be accepted to re-accumulate over time in the posterior semi-circular canal, causing repeat symptoms. In this situation, it is quite likely that patients will re-present to a clinician [12].

Residual dizziness may be present after canalith repositioning procedures (CRP), particularly in the context of our findings that vestibular rehabilitation therapy (VRT) was more effective in improving Vertigo Symptom Scale–short form (VSS–sf) scores of diabetic patients with BPPV.

Residual dizziness after successful CRP is a known phenomenon that has been reported in the literature. Martellucci et al., (2022) found that regular daily physical activities play a crucial role in preventing residual dizziness after CRP [23,28]. Their study demonstrated that patients who returned to their regular physical activities after CRP had a significantly lower incidence of residual dizziness compared to those who did not. This finding highlights the importance of incorporating physical activities in the management of patients with BPPV, especially after CRP, to reduce the occurrence of residual dizziness.

In our study, we found that VRT was more effective in improving VSS–sf scores compared to Epley–CRP. This suggests that VRT may be a more appropriate treatment option for diabetic patients with BPPV, as it not only addresses the mechanical problem of displaced otoconia, but also targets the underlying balance deficits that may be present in diabetic patients. Furthermore, VRT can be tailored to individual patients, which may contribute to better treatment outcomes.

Considering the findings of Martellucci et al., (2022) in the context of our study, it is possible that the incorporation of physical activities, as seen in VRT, may have contributed to its superiority in reducing dizziness in diabetic patients with BPPV. Therefore, the integration of physical activities in the management of BPPV, particularly after CRP, may help in minimizing residual dizziness and improving the overall treatment outcomes for these patients.

In conclusion, our study supports the use of VRT as a more effective treatment option for diabetic patients with BPPV compared to Epley–CRP. The incorporation of physical activities, as suggested by Martellucci et al., (2022), may further enhance the effectiveness of VRT in reducing residual dizziness and improving the overall quality of life for patients with BPPV.

Thus, VRT is a more effective treatment option than Epley–CRP in diabetic patients with BPPV. Healthcare providers should consider VRT as the first-line treatment for these patients to improve their balance and quality of life. Whilst this study was found to be helpful in many ways, it does have some drawbacks, such as a lack of long-term follow-up and trials with a bigger sample size, which can be addressed in future research.

## 5. Limitations of Study

In this study, there are some limitations that need to be addressed. Firstly, the sample size of 30 subjects, although modest, provides preliminary insights that can guide future research on the topic. Secondly, the purposive sampling method employed in the study, while not ideal for generalisation, allows for the selection of specific participants, which can yield valuable insights for hypothesis generation. Thirdly, the 4-week treatment duration, while not capturing long-term effects, provides a snapshot of the interventions’ immediate impact, serving as a foundation for future longitudinal studies. Fourthly, this study offers valuable insights into the recovery of BPPV using self-reported symptom scales—namely, the VSS–sf and BSS—but it falls short in providing a comprehensive assessment of BPPV recovery due to the lack of detailed information on nystagmus evaluation. Future research should address this limitation by incorporating both objective and subjective measures, allowing for a more complete understanding of BPPV recovery and its contributing factors. This would ultimately contribute to the development of more effective treatment strategies and improved patient outcomes.

Moreover, the assessor’s blinding in the study minimised assessment bias, although further studies may benefit from incorporating participant and therapist blinding, to mitigate potential biases further. In the absence of a control group receiving no intervention, the comparison between the Epley–canalith repositioning procedure and vestibular rehabilitation therapy interventions still offers meaningful insights into their relative effectiveness. Finally, while the study does not directly address potential confounding factors such as diabetes duration, glycemic control, or other comorbidities, these factors can be considered in future study to refine the understanding of the interventions’ effectiveness.

## 6. Conclusions

The study findings suggest that both the Epley–canalith repositioning procedure and vestibular rehabilitation therapy are beneficial and efficient in the management of BPPV in diabetic patients. Although vestibular rehabilitation therapy shows a trend towards greater improvement in Berg Balance Scale scores, the difference between the two treatments is not statistically significant. Therefore, it cannot be concluded that vestibular rehabilitation therapy is superior to the Epley–canalith repositioning procedure.

Nevertheless, vestibular rehabilitation therapy can be considered a valuable rehabilitation technique for physiotherapists to use in their daily practice for improving vertigo, postural stability, and activities of daily living in diabetic patients with BPPV. However, the results of this study should be interpreted with caution due to its limitations, including a small sample size and lack of gender-based analysis. Further research is needed to confirm these findings and compare the effectiveness of these treatments to other modalities.

## Figures and Tables

**Table 1:** Protocol for habituation exercises [11,12].

Sequence		Direction	Change of Position	
S1	S2		From	To
M1		Mi	Sitting	Supine
M2		Le	Supine	Left side
M3		Ri	Supine	Right side
M4		Mi	Supine	Sitting
	M5		Turning to the right	
	M6		Turning to the left	
M7		Ri	Nose on left knee	Right ear on right shoulder
M8		Le	Nose on right knee	Left ear on left shoulder
	M9		Turning head counter-clockwise	
	M10		Turning head clockwise	
	M11		Bending forward	
	M12		From sitting to erected standing position	
	M13		Moving head and backward	
M14		Le	Sitting	Head hanging and turning to the left
M15		Le	Sitting	Head hanging and turning to the left
M16		Ri	Sitting	Head hanging and turning to the right
M17		Ri	Sitting	Head hanging and turning to the right
M18		Mi	Sitting	Head hanging and turning to the right
M19		Mi	Sitting	Head hanging and turning to the left

S1, Series I: manoeuvres with possible nystagmus; S2, Series II: manoeuvres in which nystagmus never occurred; M1–19: sequence of the manoeuvres; Mi: manoeuvre executed in middle; Le: manoeuvre directed to the left; Ri: manoeuvre directed to the right.

**Table 2:** Gaze stability and balance exercises in different positions.

A. In bed	Eye movements, at first slow, then quick: (a) Up and down; (b) From side to side; (c) Focus on finger moving from 3 ft to 1 ft away from the face. Head movements, at first slow, then quick with eyes opened, later with eyes closed: (a) Bend backwards and forwards; (b) Turn from side to side.
B. Sitting	1. As above; 2. As above; 3. Shoulder shrugging and circling; 4. Bend forward and pick up object from the ground.
C. Standing	1. As A1, A2, and B3; 2. Change from sitting to standing with eyes open, then eyes closed; 3. Throw a small ball from hand to hand (above eye level); 4. Throw a small ball from hand to hand under the knee; 5. Change from sitting to standing and turn around.

**Table 3:** Demographic characteristics (Mean ± SD) of two groups.

Variable	Group A (*n* = 15) (%)	Group B (*n* = 15) (%)	*t*/χ^2^ Value	*p* Value
Age (years): Mean ± SE	39.80 ± 3.89	39.00 ± 2.69	0.17	0.867
Gender:				
Female	6 (40.0)	5 (33.3)	0.14	0.705
Male	9 (60.0)	10 (66.7)		
Dix–Hallpik3 test:				
Positive nystagmus present	15 (100.0)	15 (100.0)	0.00	1.000

Group A: Epley–Canalith Repositioning Procedure, Group B: Vestibular Rehabilitation Therapy.

**Table 4:** Pre-treatment to post-treatment change in outcome measurement scores (Mean ± SD) within the two groups.

Outcome Measures	Group	Pre (*n* = 15)	Post (*n* = 15)	Mean Change (Pre–Post)	*t* Value	*p* Value
VSS–sf score	Group A	38.93 ± 0.93	8.80 ± 0.83	30.13 ± 1.43	21.06	<0.001
	Group B	38.87 ± 1.32	4.00 ± 0.46	34.87 ± 1.52	22.97	<0.001
BBS Score	Group A	18.80 ± 0.64	43.40 ± 0.69	24.60 ± 1.07	22.93	<0.001
	Group B	23.13 ± 1.26	49.07 ± 1.22	25.93 ± 1.70	15.23	<0.001

VSS–sf: Vertigo symptom scale–short form; BBS: Berg Balance Scale; Group A: Epley–Canalith Repositioning Procedure; Group B: Vestibular Rehabilitation Therapy.

**Table 5:** Pre-treatment to post-treatment change in outcome measurement scores (Mean ± SD) between two groups.

Outcome Measures	Group	Mean Change (Pre–Post)	*t* Value	*p* Value
VSS–sf score	Group A	30.13 ± 1.43	2.27	0.031
	Group B	34.87 ± 1.52		
BBS Score	Group A	24.60 ± 1.07	0.66	0.513
	Group B	25.93 ± 1.70		

VSS–sf: Vertigo symptom scale–short form; BBS: Berg Balance Scale; Group A: Epley–Canalith Repositioning Procedure; Group B: Vestibular Rehabilitation Therapy.

## Data Availability

The datasets analyzed in the current study are available from the corresponding author on reasonable request.
